# Does Function Determine the Structure? Changes in Flexor Hallucis Longus Muscle and the Associated Performance Related to Dance Modality: A Cross-Sectional Study

**DOI:** 10.3390/medicina56040186

**Published:** 2020-04-16

**Authors:** Blanca De-la-Cruz-Torres, Irene Barrera-García-Martín, Mónica De la Cueva-Reguera, María Bravo-Aguilar, María Blanco-Morales, Emmanuel Navarro-Flores, Carlos Romero-Morales, Vanesa Abuín-Porras

**Affiliations:** 1 Department of Physiotherapy, University of Seville, Avicena Street, 41009 Sevilla, Spain; bcruz@us.es (B.D.-l.-C.-T.); irebargar@gmail.com (I.B.-G.-M.); 2 Faculty of Sport Sciences, Universidad Europea de Madrid, Villaviciosa de Odón, 28670 Madrid, Spain; monica.delacueva@universidadeuropea.es (M.D.l.C.-R.); maria.bravo@universidadeuropea.es (M.B.-A.); maria.blanco@universidadeuropea.es (M.B.-M.); vanesa.abuin@universidadeuropea.es (V.A.-P.); 3 Frailty Research Organized Group (FROG), Department of Nursing, Faculty of Nursing and Podiatry, University of Valencia, 46010 Valencia, Spain; emmanuel.navarro@uv.es

**Keywords:** biomechanics, dancer, ultrasound, muscle size, flexor hallucis longus, physiological testing

## Abstract

*Background and Objectives*: Flexor hallucis longus pathology is one of the most common conditions of the ankle and foot in dancers, due to the high demand of dance movements performed in an extreme plantar flexion and dorsiflexion range of motion. The objectives of this study were to determine the bilateral differences between the thickness and cross-sectional area of the flexor hallucis longus muscle in dancers, to establish possible differences between dance modalities, and to analyze whether there is a correlation between ultrasonographic parameters or performance variables and the dance modality. *Material and Methods*: A sample of 50 (29 classical and 21 contemporary) full-time pre-professional female dancers were included in the study. The thickness and cross-sectional area of the flexor hallucis longus muscle were evaluated for both limbs using ultrasound imaging. The range of movement of the first metatarsophalangeal joint was measured using functional extension with maximal ankle plantarflexion, balance was measured in a unilateral stance with the heel raised, endurance was evaluated through a modified heel rise fatigue test, and a counter movement jump to assess the vertical jump performance was measured bilaterally. *Results*: There were no significant differences recorded between the dominant and non-dominant limbs for each variable, within both groups. Contemporary dancers showed a greater thickness and cross-sectional area of the flexor hallucis longus muscle than classical dancers. However, classical dancers showed an increase of balance, endurance, range of movement of the first metatarsophalangeal joint, and counter movement jump with respect to contemporary dancers. *Conclusion*: Bilateral symmetry was identified in all variables for both groups. The size and performance of the flexor hallucis longus muscle may be influenced by the specific nature of dance modality.

## 1. Introduction

Classical ballet and contemporary dance performances are as significantly different due to the physical demands exerted on their artists as the artistic aspects of the choreography. Ballet is characterized by longer periods at rest and high to very high exercise intensities, while contemporary dance is related to moderate to high exercise intensities. Therefore, the energy system employed during a ballet performance is more of an anaerobic system than that in contemporary dance. In addition, the technical gesture (en pointe) is more prevalent in classical dance compared to contemporary dance [1].

The flexor hallucis longus muscle (FHL) is considered the primary active plantar flexor of the first metatarsophalangeal (MTP) and interphalangeal (IP) joints of the hallux and limits the passive dorsiflexion at the first MTP joint [2]. In addition, the FHL muscle is a secondary plantar flexor of the ankle. The FHL tendon becomes compressed within the fibro-osseous tunnel as the muscle contracts when the dancer is en pointe (ankle plantar flexion). In contrast, the FHL tendon is stretched between the talar tubercles and sustentaculum tali when the dancer is in a demi-plié (ankle dorsiflexion) [3].

FHL tendinopathy is one of the most common conditions of the ankle and foot in dancers, due to the high demand of dance movements alternating between extreme plantar flexion and dorsiflexion [4,5]. It is a very painful and disabling condition and may be detrimental to a dancer’s career. While FHL tendinopathy is also present in non-dancers, the condition is more severe in dancers, with a three times longer duration of symptoms and 71% incidence of tendon tears (versus 30% in non-dancers) [6].

Ultrasound imaging (USI) has been employed to assess the thickness and cross-sectional area (CSA) of many different musculoskeletal structures [7,8,9,10]. Understanding the relationship between the lower leg muscle’s structure and performance is essential to explaining how dance activities may relate to changes in foot function. Furthermore, the characterization of individual foot structures is required to explain the structural and functional adaptation of the foot to the sport, in this case, dance.

Ultrasound imaging is a non-invasive, safe, and valid method for examining soft tissue and musculoskeletal structures [11,12]. Prior studies have focused on the thickness and CSA of the FHL muscle in healthy subjects [13] and subjects with pes planus [14]. Recently, Shih et al. [15] found that the FHL tendon CSA was significantly larger in dancers than in non-dancers. However, there is limited evidence regarding the relationship between surrounding soft tissues and adaptations to sports or performance. Characterization of the FHL muscle morphology as it relates to flexibility, strength, and function in young dancers is required as a foundation for focused investigations on performance and injury prevention strategies. Several authors have related muscle morphology changes measured by USI to muscle strength, altered muscular function, and pain conditions in different populations [16]. Therefore, the aims of this study were to determine whether a bilateral difference in the thickness and CSA of the FHL muscle exists in classical and contemporary pre-professional dancers, to assess the possible differences in the muscular features and ballet functional examination tests between both dance modalities, and to analyze whether there is a correlation between ultrasonographic parameters or performance variables and the dance modalities.

## 2. Material and Methods

### 2.1. Design

An observational study, adhering to the Strengthening the Reporting of Observational Studies in Epidemiology (STROBE) [17] guidelines, was performed between July and August 2019.

### 2.2. Participants

The target sample comprised 53 pre-professional female dancers from a dance school. There were three dancers lost to the study due to illness. A total of 50 fulltime pre-professional dancers (aged 15–28 years) were included in the study. The sample was divided into two groups: classical dancers (*n* = 29) and contemporary dancers (*n* = 21). The classification of dancers was based on their professional career within the dance school. Generally, they decide on a style of dance and receive classes focused on that style. All dancers performed the same dance hours per week and had the same number of years of dance training; the difference was the technique, due to classical dancers performing pointe hours per week compared to contemporary dancers (Table 1).

Male dancers were excluded from the study, as gender differences in the dance technique and training exist, and because overuse injuries most often affect female ballet dancers because they work en pointe, while males usually do not. Inclusion criteria were female classical or contemporary healthy dancers, with no acute lower limb injury in the last six months and no history of ankle surgery.

### 2.3. Sample Size

A pilot study (*n* = 8) was conducted to determine the difference in FHL muscle thickness (mean cm ± SD) between two groups of four classical (2.58 ± 0.32 cm) and four contemporary (2.34 ± 0.28 cm) dancers. G*Power software [18] was then used to calculate the sample size using a one-tailed hypothesis, an α error of 0.05, an effect size of 0.79, a power of 0.80, and an allocation ratio of 1, for the calculation. Therefore, a total sample size of 42 individuals was calculated. We managed to recruit a sample of 50 subjects.

### 2.4. Ethical Statement

The study received ethical approval from the local ethics committee (Acta06/ 19 June/2019) and complies with all the principles set out in the Declaration of Helsinki [19]. All the subjects (or parents/guardians for dancers under 18 years of age) were fully informed of the protocol, and written informed consent was obtained from each dancer or parent/guardian, prior to testing.

### 2.5. Ultrasonography Measurements

**Figure 1:**
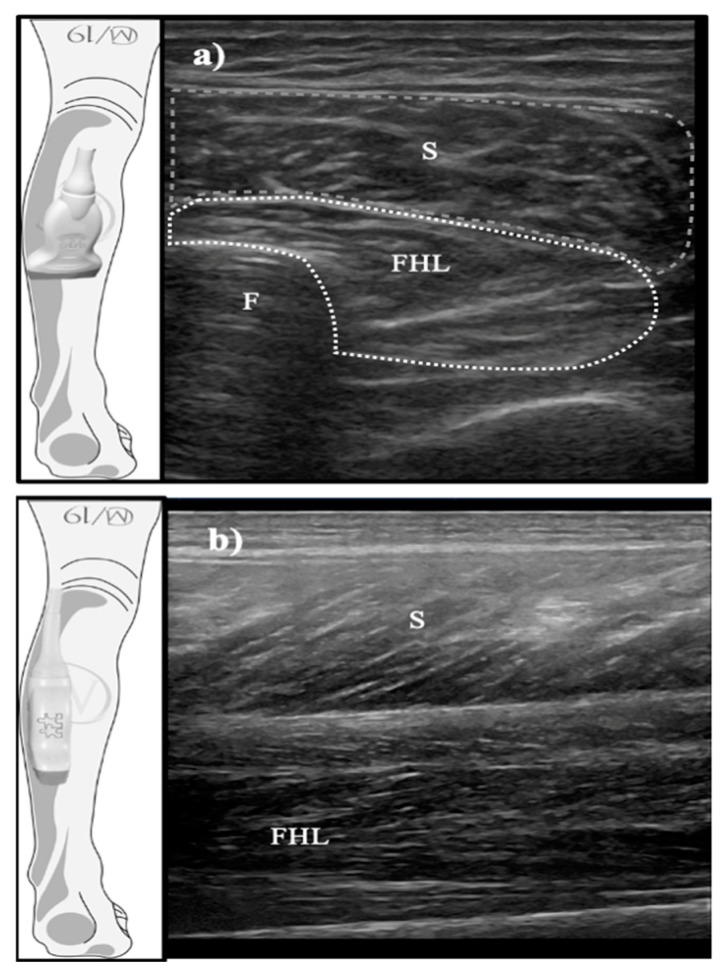
Probe position (transverse (**a**) and longitudinal views (**b**)), orientation, and sample images for the FHL muscle. FHL: flexor hallucis longus muscle; F: fibula; S: soleus muscle.

A physiotherapist with 10 years of experience in ultrasonography carried out all of the examinations. The dancers lay prone with their feet off the table [20,21]. As previously described [7,8], the FHL muscle was located, longitudinally and transversally, at 50% of the distance between the fibular head and inferior border of the lateral malleolus on the posterior aspect of the fibula using an ultrasound machine (Logiq, GE Healthcare: Chicago, IL, USA) with a 12 MHz linear transducer. In the ultrasound image, the CSA was defined as the area of the cross-section of the FHL muscle perpendicular to its longitudinal dimension, and the thickness was considered as the distance between aponeuroses. The correct position and orientation of the probe are in shown in Figure 1. The final scores were calculated using the mean of three repeated values for each measurement with the ImageJ software (Bethesda, MD, USA).

### 2.6. Ballet Functional Examination

As previously described in the scientific literature [15,22,23], four tests were conducted in the following order: range of movement (ROM) of the first MTP joint, a balance test, an endurance test, and the vertical jump performance. All physical measurements were obtained by a trained physiotherapist who was blinded to each subject’s group allocation.

Range of movement of the first MTP joint: Evaluated using functional MTP joint extension with maximal ankle plantar flexion in a seated position. The ROM was measured using a universal goniometer.

Balance test: Dancers balanced in a unilateral stance (demi-pointe position) with their heel raised and eyes open, until the point of imbalance (when they applied full foot support on the floor).

Endurance test: A modified unilateral heel raise fatigue test was performed on a stable block, with the participant’s toes over the edge of the block. Heel raises were performed to a metronome tempo of 30 bpm. A light touch on an adjacent wall was permitted, to assist with balance. The number of heel raises completed until fatigue, defined by the failure to maintain the tempo or heel height for at least three consecutive raises, was recorded. According to Rowley [22], this test was performed with “toes off the edge”, because the standard heel performance depends on active control of the toes.

Vertical jump (VJ) performance was also measured. This is a widely used marker of physical performance in athletes [24], including dancers [23]. A counter-movement jump (CMJ) is a common jumping task used to measure the VJ performance [25]. Each participant performed three practice trials of the CMJ. The CMJ was performed with the participant’s hands on their hips and starting from a static position with the legs straight. Participants were instructed to squat by bending at the knees, then jump as high as possible, keeping the legs straight, and land with both feet together. Two attempts were performed, with a two-minute passive rest between attempts. The counter-movement jump was measured by the Myjump App on iPhone 6S (Apple: Shenzen, China) [26].

All measurements were recorded for the dominant and non-dominant limb. The non-dominant limb was considered the dancer’s preferred limb for balancing whilst gesturing with the contralateral limb. The dominant limb was defined as the preferred kicking limb. All testing was performed barefoot.

### 2.7. Statistical Analysis

First, Kolmogorov–Smirnov analysis and Levene’s tests were conducted to assess the data normality and the homogeneity of variance across groups, respectively. Second, descriptive statistics were carried out for the two groups. Third, a comparative analysis was conducted within each dance modality group to distinguish appreciable differences between each dancer’s limbs, followed by an analysis of the two groups. The mean and standard deviation (SD) using Student’s *t*-test were employed for parametric data, and the mean and interquartile range (IR) were used to describe the non-parametric data. Magnitude-based inferences on the differences between the dance modality (classical versus contemporary dance) and limb (dominant versus non-dominant) were determined using a spreadsheet designed by Hopkins et al. [27] for change scores between paired comparisons for each variable. The standardized difference or effect size (ES, 90% confidence limit [90% CL]) in the selected variables was calculated. Threshold values for assessing magnitudes of the ES (changes as a fraction or multiple of baseline standard deviation) were >0.20, 0.20, 0.60, 1.2, and 2.0 for trivial, small, moderate, large, and very large, respectively [27]. The qualitative magnitude of beneficial/better or detrimental/poorer effects was assessed qualitatively as follows: <75%, unclear; >75–95%, likely; >95–99%, very likely; and >99%, almost certainly. A substantial effect was set at >75% [28]. Finally, a univariate analysis was performed using a linear regression (stepwise selection method; Pin = 0.05; Pout = 0.10), in order to predict the influence of the group on the statistically significant outcome measurements (presented in the prior described analysis). The independent variables were the thickness and CSA of the FHL muscle, endurance test, balance test, ROM of the first MTP joint, and CMJ. The analysis was completed using the Statistical Package for Social Sciences (SPSS) (v. 21.0 for IOS IBM Corp) and an online spreadsheet (www.sportsci.org).

Finally, the intra-observer reliability was calculated using the intraclass correlation index (ICC) [29] for the ROM, thickness, and CSA.

## 3. Results

Fifty female dancers (100 limbs) were recruited for the study. There were 29 classical ballet dancers (58 limbs) and 21 contemporary dancers (42 limbs). There were no significant baseline differences between groups for any participant characteristics, including the age, weight, height, body mass index, dance hours per week, and years of dance training (all *p* > 0.05) (Table 1).

Table 2 shows the descriptive values or results for the thickness and CSA of the FHL muscle, balance and endurance tests, ROM of the first MTP joint, and CMJ for both limbs, for classical and contemporary dancers, respectively. Statistical analysis reported no meaningful differences between dominant and non-dominant limbs for each variable in both groups (Table 2).

Table 3 shows meaningful differences between dancers. Contemporary dancers showed higher scores than classical dancers for the thickness and CSA of the FHL muscle. However, classical dancers showed higher scores than contemporary dancers for balance and endurance tests, ROM of the first MTP joint, and CMJ (Table 3).

Regarding Table 4, the prediction model for the thickness and CSA of the FHL muscle (R^2^ = 0.014, ß = 0.11 and R^2^ = 0.010, ß = 0.38, respectively), endurance test (R^2^ = 0.012, ß = 0.10), balance test (R^2^ = 0.002, ß = 0.45), ROM (R^2^ = 0.002, ß = 0.43), and CMJ (R^2^ = 0.001, ß = 0.001) was determined by group (contemporary).

Finally, the intra-observer reliability showed a high intra-examiner reliability for ROM, thickness, and CSA variables (ICC = 0.91, ICC = 0.90, and ICC = 0.95, respectively).

## 4. Discussion

In this study, no significant side-to-side differences were found between the dominant and non-dominant limb for all variables in the classical and contemporary dancers. With respect to the reference values provided by other authors, the data from this study shows that the dancers have a slightly larger value for both the thickness and CSA of the FHL muscle [7,14].

Regarding whether the repetitive loading forces generated during dance training could induce bilateral differences in the FHL muscle profile and performance in dancers, the results of this study are in line with previous research conducted among football players [30], tennis players [31], and baseball pitchers [31], which reported no significant differences (*P* > 0.05) between limbs for the ankle dorsiflexion ROM and hip rotation, respectively. Both limbs were found to be bilaterally symmetrical in those studies.

Dance requires the participants to perform repeated, high-intensity movements such as a pointe, a demi-plié, jumping, and landing. Several studies have addressed variables such as ankle dorsiflexion [32,33], excessive forces, and loading rates during landing, as risk factors for injuries in this population. The high-intensity demands of movements required in dance could lead to high development of the muscles or overload in the joints. This may generate dance-specific adaptations that could cause impairments in ROM during dance activities, which may result in either an improvement of the performance or a risk of injury [20]. It is therefore important to analyze the possible ankle and foot ROM dance-specific adaptations at a pre-professional level, in order to effectively plan and establish successful prevention and rehabilitation programs.

Rowley et al. [22] found that dancers rely on the toe flexors more than non-dancers to complete balance and heel raise tasks. Shih et al. [15] argued that the FHL tendon CSA was significantly larger in dancers than in non-dancers. However, the results of the present study found differences between groups based on the dance modality itself. Contemporary dancers had higher scores than classical dancers for the thickness and CSA of the FHL muscle. This difference can be attributed to different factors, as the two modalities include different training and different equipment. Classical dancers use special reinforced footwear for training and performance. This special footwear includes a toe cap that may act as a stabilizer of the MTP joint and thus decrease the intensity of the activity demands from the FHL muscle [34]. Nevertheless, classical dancers showed higher scores than contemporary dancers for the balance and endurance tests, ROM of the first MTP joint, and CMJ. Therefore, it may be hypothesized that these differences are attributed to the dancer’s training effect, since classical dancers adopt the en pointe position more than their contemporary counterparts. This en pointe position is highly challenging for balance, strength, and endurance, which may explain this difference between groups [35].

### Clinical Relevance and Future Lines

The findings of the present study showed differences related to training and performance in parameters of muscular thickness of the FHL, ROM, and ballet performance tests. This may be relevant to clinical practice, as some form of “cross-training” could be designed for one dance modality group to avoid the adaptations associated with only performing one type of dance.

Some authors have addressed the changes in thickness in the tendon and muscle complex in the foot in the presence of tendinopathy [8,36]. Therefore, ultrasonography signs of changes in the FHL thickness could be an early warning of tendon pathology development and promote the implementation of prevention and even early treatment protocols. The screening of FHL muscle for dancers should be conducted during the season (at the beginning, in the middle, and at the end), and eccentric training should mainly be prescribed to dancers to lower the risk of developing a muscle injury. Sammarco and Cooper [6] carried out a study which showed that dancers tended to have symptoms of FHL tendon injury for a longer time before demanding treatment than non-dancers. Therefore, dancers, as a high-risk group, should benefit from early assessment regarding the FHL state.

The study presented several limitations. Firstly, the dancer’s training load was not analyzed, which may have influenced, or correlate to, the studied variables. Secondly, other muscles were not included in the study. This may have been useful to complete the analysis of the effect of muscle work on the performance in the tests.

Future lines of research should address the lack of evidence regarding effective injury prevention programs for FHL pathology in dancers, considering the high incidence of this condition within this population. Further research on FHL pathology, endurance, and balance between different types of “contemporary” dance is still needed. Moreover, in order to eliminate the bias of user-dependent US examination, further studies will be developed using MRI.

## 5. Conclusions

In conclusion, this study identified bilateral symmetry in all studied variables for both groups. The results showed a greater CSA and thickness in the FHL muscle for the contemporary dancers, and greater balance and endurance, ROM of the first MTP joint, and CMJ height for the classical dancers. These findings might reflect possible specific adaptations dependent on the dance modality.

## Figures and Tables

**Table 1:** Participant characteristics and comparative *p* values for the two groups.

	Classical Ballet Group (*n* = 29)	Contemporary Group (*n* = 21)	*P* Value
Age (years)	17 ± 2.00 (16.00–18.50) ^†^	17 ± 3.50 (16.50–20.00) ^†^	0.910 ^‡^
Weight (kg)	53.48 ± 4.64 (42.00–60.00) *	57.56 ± 6.32 (45.00–68.00) *	0.173 ^**^
Height (m)	1.66 ± 0.50 (1.55–1.76) *	1.65 ± 0.06 (1.54–1.79) *	0.131 ^**^
BMI (kg/m^2^)	19.39 ± 1.52 (17.10–22.04) *	21.16 ± 2.24 (16.53–24.91) *	0.731 ^**^
Dance training (years)	10.55 ± 3.04 (4.00–15.00) *	10.60 ± 3.01 (5.00–18.00) *	0.957 ^**^
Dance hours/week	22.00 ± 7.50 (14.50–22.0) ^†^	22.00 ± 1.75 (21.00–22.75) ^†^	0.875 ^‡^
Pointe hours/week	8.00 ± 5.50 (4.50–10.00) ^†^	N/A	N/A

Abbreviations: BMI, body mass index. * Mean ± standard deviation (minimum-maximum) was applied. ** Student’s *t*-test for independent samples was performed. ^†^ Median ± interquartile range (25th percentile; 75th percentile) was used. ^‡^ Mann–Whitney *U* test was utilized.

**Table 2:** Relative differences and qualitative outcomes in thickness and CSA of the FHL muscle, balance test, endurance test, ROM of the first MTP, and CMJ between the dominant and non-dominant limb for each group.

	Classical Dancers				Contemporary Dancers			
	Dominant Limb	Non-Dominant Limb	ES (CL)	Qualitative Magnitude	Dominant Limb	Non-Dominant Limb	ES (CL)	Qualitative Magnitude
Thickness FHL (cm)	2.48 ± 0.42	2.42 ± 0.29	0.08 ± 0.38	29/59/11 Unclear	2.62 ± 0.36	2.64 ± 0.31	0.03 ± 0.28	16/76/9 Unclear
CSA (cm^2^)	3.86 ± 0.87	3.90 ± 0.96	−0.01 ± 0.39	18/62/21 Unclear	4.05 ± 0.79	4.18 ± 0.88	0.10 ± 0.26	26/71/3 Unclear
Balance test (sec)	22.13 ± 12.63	25.03 ± 14.37	−0.25 ± 0.49	6/37/57 Unclear	13.35 ± 8.46	17.24 ± 12.61	−0.20 ± 0.18	0/51/48 Unclear
Endurance test (rep)	24.90 ± 6.48	26.34 ± 6.25	−0.12 ± 0.43	11/52/37 Unclear	22.38 ± 7.49	22.00 ± 6.84	−0.18 ± 0.42	7/46/47 Unclear
ROM 1° MTP (°)	101 ± 4.01	101 ± 4.84	0.34 ± 0.62	66/27/7 Unclear	93 ± 6.37	93 ± 6.98	0.30 ± 0.39	67/31/2 Unclear
CMJ (cm)	10.44 ± 2.47	10.67 ± 2.45	−0.15 ± 0.52	13/44/43 Unclear	9.32 ± 1.89	9.71 ± 2.42	0.14 ± 0.62	43/39/18 Unclear

Data are reported as the mean ± standard deviation (SD). CL = confidence limits; ES = effect size; Chances = percentage chance of having better/similar/poorer values; CMJ: counter-movement jump; CSA: cross-sectional area; FHL: flexor hallux longus; MTP: metatarsophalangeal; ROM: range of motion. ^*^ Student’s *t*-test for independent samples was performed (*p* > 0.5).

**Table 3:** Relative differences and qualitative outcomes for the thickness and CSA of the FHL muscle, balance test, endurance test, ROM of the first MTP, and CMJ between classical and contemporary dancers.

	Classical Dancers (58 limbs)	Contemporary Dancers (42 limbs)	ES (CL)	Chances (%)	Qualitative Magnitude
**Thickness FHL (cm)**	2.45 ± 0.36	2.62 ± 0.33	0.37 ± 0.35	79/21/0	Likely *
**CSA FHL (cm^2^)**	3.83 ± 0.90	4.26 ± 0.87	0.57 ± 0.36	95/5/3	Very likely *
**Balance test (sec)**	23.58 ± 13.49	14.01 ± 10.06	−0.88 ± 0.32	0/0/100	Almost certainly **
**Endurance test (repetitions)**	25.62 ± 6.35	22.19 ± 7.09	−0.64 ± 0.43	0/5/95	*Very likely* ^†^
**ROM 1° MTP (°)**	101 ± 4.83	93 ± 6.42	−1.37 ± 0.38	0/0/100	*Almost certainly* ^†^
**CMJ (cm)**	10.56 ± 2.44	9.52 ± 2.15	−0.43 ± 0.38	0/15/85	Likely *

Data are reported as the mean ± standard deviation (SD). CL = confidence limits; ES = effect size; Chances = percentage chance of having better/similar/poorer values; CMJ: counter-movement jump; CSA: cross-sectional area; FHL: flexor hallux longus; MTP: metatarsophalangeal; ROM: range of motion. Student’s *t*-test for independent samples was performed (*) *p* < 0.05; (**) *p* = 0.01; (^†^) *p* < 0.001.

**Table 4:** Multivariate predictive analysis for the thickness and CSA of the FHL muscle, endurance, balance, ROM, and CMJ variables determined by group.

Parameter	Model *P* Value	*R*^2^ Change	Beta Coefficient 95% CI (Min–Max)
FHL thickness	2.424	0.010	0.38 (−0.173–0.225)
	−0.26 * Group		
	*P* = 0.795		
FHL CSA	3.794	0.014	0.11 (−0.735–0.304)
	−2.15 * Group		
	*P* = 0.409		
Endurance	26.345	0.012	0.10 (−5.298–2.418)
	−1.440 * Group		
	*P* = 0.457		
Balance	25.025	0.002	0.45 (−9.295–6.782)
	−1.256 * Group		
	*P* = 0.775		
ROM	101.48	0.002	0.43 (−2.453–3.297)
	−0.422 * Group		
	*P* = 0.679		
CMJ	10.667	0.001	0.01 (−1.489–1.302)
	−0.093 * Group		
	*P* = 0.894		

*Abbreviations*: CMJ, counter movement jump; FHL, flexor hallucis longus; ROM, range of motion; * Multiplay: Group (classic = 0; contemporary = 1); sex (women = 0; men = 1).
